# Micronutrient interventions among vulnerable population over a decade: A systematic review on Indian perspective

**DOI:** 10.34172/hpp.2022.19

**Published:** 2022-08-20

**Authors:** Soorya Haridas, Jancirani Ramaswamy, Tharanidevi Natarajan, Prema Nedungadi

**Affiliations:** ^1^Department of Sciences, Amrita School of Physical Sciences, Amrita Vishwa Vidyapeetham, Coimbatore, Tamil Nadu, India; ^2^Amrita Create, Amrita School of Computing, Amritapuri, Amrita Vishwa Vidyapeetham, Kerala, India

**Keywords:** Micronutrients, Deficiencies, Hidden hunger, Malnutrition, Anemia

## Abstract

**Background:** Micronutrient deficiency has long been recognized as a public health problem, particularly among vulnerable groups such as children, adolescents, pregnant and lactating women. Micronutrient deficiency could not be ruled out in spite of the implementation of various intervention strategies. Different interventions are being used to prevent and treat micronutrient deficiencies at the national and global level. The aim of this study is to systematically review the intervention strategies among different vulnerable age groups in India.

**Methods:** The review was focused on identifying various interventions published based on the internet databases and the peer-reviewed papers from 2011 to 2021, on the predefined inclusive/exclusive criteria. The major intervention strategies implemented in India were recognized and evaluated based on dietary supplementation, micronutrient supplementation, knowledge interventions and food fortification among various age groups.

**Results:** The results show that there are still considerable gaps in identifying the effective intervention strategies, research initiatives, programs and policies addressing to tackle micronutrient deficiencies in India. Multiple interventions are effective that could lead the road to innovations in approaches with diverse dietary intake, developing multiple micronutrient supplements, fortifying foods and nutrition interventions to address calcium, zinc, iodine, vitamin D and vitamin A deficiencies among the vulnerable population.

**Conclusion:** Evidence-based multiple intervention studies covering a large population, in the long term cross-sectional, is the need for the hour to design policies and programs for improving the micronutrient status of vulnerable population in the community.

## Introduction

 Micronutrient deficiencies are a critical global health concern is taking a toll on the vulnerable population including infants, preschoolers, adolescents, pregnant women, lactating mothers and the aged population.^1^ Micronutrient deficiencies are amongst the highest risk driving variables for morbidity and poor quality of life among the vulnerable groups.^2^ The term “hidden hunger” is used to explain “chronic micronutrient deficiencies” as they mainly exist in children who are not grouped as malnourished according to the measurements of stunting or wasting.^3^ Conferring with the World Health Organization (WHO), micro nutrient deficiencies in iodine, iron and vitamin A are global health issues and the mission is to conduct research and implement effective micronutrient programs.^4,5^ The Comprehensive National Nutrition Survey (CNNS) in India indicated a high prevalence of anemia (24–41%), iron deficiency (17–32%), folate deficiency (23–37%) and vitamin B12 deficiency (14–31%).^6^ National Family Health Survey-IV (NFHS) data show that more than half of the Indian women (53.0%) in the reproductive age group (15–49 years) are anemic (Table 1).^7,8^

**Table 1:** National and Global statistics on prevalence of micronutrient deficiencies

**Micronutrient deficiencies**	**National statistics**	**Global statistics**
Iron (IDA)	Children (6-59 months) – 59%* Preschoolers (1-4 years) – 32%** School going children (5-9 years) – 17%** Female Adolescents (10–19 years) – 31%** Male Adolescents (10–19 years) – 12%** Women (15-49 years) – 53%* Men (15-49 years) – 23%* Pregnant women – 50% Breastfeeding mothers – 58%*	Children (6-59 months) – 42%*** Women (15-49 years) – 30%*** Pregnant women – 40%***
Iodine	Women (15-49 years) – 2%* Men – < 1%* Preschoolers (1-4 years) – 4.6%** School age children (5-9 years) – 4.4%** Adolescents (10–19 years) – 5.2%**	School age children (6-12 years) – 30%*** General population – 28.5%***
Vitamin A	Preschoolers (1-4 years) – 18%** School age children (5-9 years) – 22%** Adolescents (10–19 years) – 16%**	Children (6-59 months) – 60%*** Pregnant women – 20%***
Vitamin D	Preschoolers (1-4 years) – 14%** School-age children (5-9 years) – 18%** Adolescents (10–19 years) – 24%**	General population – 72.8%***
Folate	Preschoolers (1-4 years) – 23%** School age children (5-9 years) – 28%** Adolescents (10–19 years) – 37%**	General population – > 20%***
Vitamin B12	Preschoolers (1-4 years) – 14%** School age children (5-9 years) – 17%** Adolescents (10–19 years) – 31%**	General population – 40%***
Zinc	Preschoolers (1-4 years) – 19%** School age children (5-9 years) – 17%** Adolescents (10–19 years) – 32%**	General population – 10%***

Source: * NFHS-4, 2015-16^7^; ** CNNS, 2019^6^;*** WHO, 2017.^4,5^

 Food and nutrition insecurity due to low dietary intake and less dietary diversity leads to chronic health issues like protein energy malnutrition and micronutrient shortcomings. Micronutrient deficiency among women, children and adolescents is a major crisis in the country for the comprehensive growth and development in the areas of health and nutrition. Among the micronutrient deficiencies, Iron Deficiency Anemia (IDA) prevails as the most serious communal health issue. Whereas, vitamin A and vitamin D deficiencies remain clinical issues rather than public health problems.^9^ Alleviation of micronutrient deficiencies is imperative and is globally addressed through intervention strategies that include supplementation, fortification and food diversification.^10^ Major health concerns connected to dietary inadequacy include lack of critical nutrients, protein-energy malnutrition and micronutrient inadequacies.^11^

 More interventions are being attempted at different times and in different populations. The government has successfully implemented the Weekly Mass Iron and Folic Acid (IFA) Supplementation (WIFS) Programme under the National Rural Health Mission (NRHM) through Anganwadi Centers across all states in India to most of the indigenous population.^12^ There are priority programs including supplementation of vitamin A for children 6 to 59 months, supplementation of iron and folate for women of child-bearing age, salt iodisation, supplementation of zinc as a treatment for diarrheal diseases, calcium intake among adults, multiple micronutrient powders, behaviour-centred nutrition education, staple food fortification and bio-fortification of crops.^13^

 Micronutrient deficiency could not be ruled out in spite of the implementation of various intervention strategies. Considering this gap, this literature search aimed at identifying the effective intervention strategies, research initiatives, programmes and policies addressing to bring down micronutrient deficiencies among different age groups in India.

## Materials and Methods

 A methodical review was analyzed with the studies on micronutrient interventions among vulnerable age groups (infants, preschoolers, school-going children, adolescents, adults, pregnant women and lactating mothers) over the decade of 2011–2021. A systemic online search was conducted to collect research papers, review papers, reports and editorials on micronutrient intervention in India. The electronic databases were searched for relevant research in peer-reviewed journals including Scopus and Google scholar accessed from 1^st^ May 2021 to 31^st^ July 2021. The investigator does not have any direct contact with the authors for their related research. The investigator is a research scholar pursuing research in the field of micronutrient interventions. The electronic search strategy based on the Scopus database was searched using the keywords and limited to the year of research. The original articles were selected and scrutinized based on the micronutrient intervention studies and the vulnerable groups. A hierarchical search procedure was employed using the combination of keywords: (1) Micronutrient interventions- vulnerable groups, (2) Government Interventions- India, (3) Food-based approaches, (4) Dietary/fortified/micronutrient supplementations- India- adolescents- pregnant women- lactating mothers- children, (5) Knowledge Interventions- Methods/Tools-vulnerable groups-India and (6) Multiple interventions

###  Selection criteria

 Studies were short-listed and research articles were extensively evaluated based on the criteria for inclusion and exclusion. The search was restrained by age, gender, study design and type of intervention. All the peer- reviewed journals relevant to the search keywords and those with intervention studies were included. Studies published in languages other than English and before 2011 were omitted. Only studies with respect to Indian perspective, irrespective of the region and population, were included. Review studies, editorials, short communications, blogs, newsletters etc., were excluded. The selected studies were further consolidated and interpreted. Based on the literature search and the type of intervention identified, the consolidated papers were further grouped by considering the age groups, study design, mode and period of intervention.

###  Type of studies

 Thestudy designs comprised in the review were randomized controlled trials, efficacy trials, cross-sectional studies, knowledge attitude practices and comprehensive survey models. Comparative study designs were also included based on the age groups, gender and type of interventions.

###  Target population

 The target population included infants, preschoolers, school-going children, adolescents, adults (male and female), pregnant women and lactating mothers who were undergoing various micronutrient interventions around India.

###  Type of intervention

 Food-based approach is an identified sustainable strategy to combat micronutrient deficiency in the community. This includes the intervention methodologies in the vein of dietary food supplementations (value-added foods, convenience food, synergic foods and micronutrient enriched foods) for various vulnerable populations based on their nutritional status. Integrating with it intervention through fortified food supplementation (fortified and biofortified foods) is also one of the approaches to tackle micronutrient deficiencies. Intervention through micronutrient supplements (tablets, syrup, powders etc.,) paved the way for preventing micronutrient deficiencies. Studies with respect to micronutrient supplements for the vulnerable population were categorized under micronutrient supplementation. Community-based approach is a supportive intervention that could bring behavioural change in knowledge, attitude and practices. This category comprises intervention strategies including nutrition education, awareness building programs, children/parental counselling etc., aiming at disseminating knowledge on better eating practices to bring out a behavioural change in the community. The methodologies/tools for the community-based approach include Information Communication Technology (ICT) tools, Information Education Communication (IEC) materials and participatory learning methods. The multiple intervention strategies include the combined impact of two or more interventions which led to more substantial results.

## Results

 Various national and international intervention programmes have been designed and implemented to enhance the micronutrient status of the population. In this review, it has been attempted to examine intervention studies that are used to alleviate micronutrient malnutrition in the Indian context. In view of the fact that micronutrient deficiencies occur in individuals of all ages, genders and regions. In most of the studies, adolescents, pregnant women, pre-school and school-going children were deemed the main targets for intervention. The selected studies are consolidated and discussed below.

###  Micronutrient intervention for infants

 Micronutrient interventions in infancy have been seen as critical in enhancing cognitive development in children. A few intervention trials examined the immediate and sustained impacts of interventions on cognitive, language, motor, behavioural development, well-being or growth in this age group as listed in Table 2.

**Table 2:** Micronutrient intervention for infants

**Target population**	**Study design **	**Intervention**	**Findings**
**Food based approach -fortified food supplementation**			
Preterm neonates	RCT	Fortified human milk (112 mg Ca & 59 mg P) for 8 months	The levels of serum calcium and phosphate in the fortified group were significantly higher.^14^
Children (0–35 months)	CSS	Fortified Salt (15 ppm I) & Fortified Rice (60 mg Fe)	Fortified rice is an effective strategy to improve the iron status & increasing the adequacy of iodization.^15^
Children (6–18 months)	Cluster-RET	Fortified MNP (12·5 mg Fe, 5 mg Zn, 0·16 mg folic acid, 0·3 mg Vit A, 30mg Vit C, 0·9 μg Vit B12, 90 μg I) for 12 months	Improved the motor and mental development.^16^
Children (6-24 months)	CRCT- (iron & zinc)	Fortified rice (7.9 mg Fe & 6.5 mg Zn/20 g sachet)	Fortified complementary food had a suitable delivery mechanism for iron and zinc.^17^
**Micronutrient supplementation**			
Infants (4 months)	RDBPCT	Zinc (5 mg) for 190 days	Significant effect on the skin fold thickness of the infants.^18^
Children (6 months)	RDBPCT	Vitamin A (50 000 IU) for 2 years	Reduce the risk of mortality.^19^
**Multiple intervention strategies- food based approach & community based approach**			
Infants	RDBPCT	*Fortified food-* Multi Micro Nutrient Powder (8-13 mg Fe, 200 µg Vit A, 20 mg Vit C, 20µg folic acid, 5 mg Zn, 0.5 µg Vit B12 & 0.5 mg Vit B2) *Nutrition education*- Multi micronutrients	MNP and learning trial has a positive effect on the health and nutrition of the children.^20^

Abbreviations: RCT, randomized controlled trial; CRCT, cluster randomized controlled trial; RDBPCT, randomized, double-blind, placebo-controlled trial; CSS, cross-sectional study; RET, randomized efficacy trial; MNP, micronutrient powder.

####  Food based approach -fortified food supplementation

 The RCT trial executed in Haryana through fortified breast milk among neonates resulted in increased serum calcium and phosphate levels.^14^ A sustainable food based cross-sectional study (CSS) through Integrated Child Development Scheme (ICDS) supplementing iodized salt and fortified rice improved the overall micronutrient status of the children (0-35 months) in Telangana.^15^ The cluster randomized efficacy trial (CRET) trial implemented in Bihar showed home fortification with micronutrient powder (MNP), distributed through the existing health care service systems for a period of 12 months had an impact on gross motor, language and personal-social development of children aged 6–18 months.^16^ The cluster randomized sample undertook intervention of complementary foods using fortified rice resulted in a significant increase in haemoglobin levels among 6 months old children in Delhi to restore the iron gaps during the breastfeeding phase.^17^

####  Micronutrient supplementation 

 The randomized intervention trial for 190 days showed a significant effect of zinc supplementation (5 mg) on linear growth and body weight gain in infants compared to the placebo group.^18^ Neonatal supplementation with vitamin A (50 000 IU) for 2 years resulted in 10% reduction in mortality in two districts of Haryana compared to the placebo receivers.^19^

####  Multiple intervention strategies- food-based approach & community-based approach

 An randomized double-blind placebo-controlled trial integrated approach through fortified MNP and early learning trial has been effective in collaboration with the ICDS programme in Hyderabad. The knowledge gained during the development, design and implementation of the double-blind trial can be used to guide large-scale policy and programs.^20^

###  Micronutrient intervention for preschoolers & school-going children

 Micronutrient deficiency is a major concern for children, who have a higher nutritional need due to physical growth and intense physical activity. This section includes intervention studies related to preschoolers and school going children. Micronutrient interventions for this vulnerable age group is consolidated, tabulated and presented in Table 3.

**Table 3:** Micronutrient intervention for preschoolers & school going children

**Target population**	**Study design**	**Intervention**	**Findings**
**Food based approach -dietary food supplementation**			
Preschoolers (2-5 years old)	RCT	Guava (1.5 mg Fe & 6.8 mg Vit C) for 6 months	Improves the iron status.^21^
School children (7-9 years)	RCT	60 g Garden cress seeds (calcium, iron and zinc) for 3 months	The effect of garden cress as additional food is a successful strategy in fighting malnutrition and anaemia.^22^
Children (5-7 years)	RCT	Fortified Salt (10 mg Fe, 400 µg I, 4 µg Vit B12, 100 µg folic acid & 10 mg Zn) for 8 months	There was a significant decline in the prevalence of anaemia and zinc deficiency with no change in iron deficiency anaemia.^23^
**Food based approach -fortified food supplementation**			
Preschoolers (4-6 years)	DBRCT	Biofortified wheat (20 ppm Zinc) for 6 months	Higher zinc content improved the morbidity status.^24^
**Micronutrient supplementation**			
Pre-pubertal School Girls (6-12 years)	CRCT	600 IU -2000IU Vit D for 5 months	Improved vitamin D sufficiency.^25^
Pre menarche girls (8-12 years)	DBCRCT	500 mg Ca, 15 mg Zn & 30000 IU Vit D for 1 year	Improved the bone mineral content.^26^
**Multiple intervention strategies- food based approach & & micronutrient supplementation**			
School going children (9 to 12 years)	RCT	*Dietary food-* Sorghum *Supplements-* (100 mg Fe, 500 µg folate & 500 mg Ca) for 8 months	Hemoglobin, serum folic acid, albumin, retinol binding protein, ferritin, calcium and iron improved.^27^
**Multiple intervention strategies- food based approach & community based approach**			
Preschoolers	RDBPCT	*Fortified food-* Multi Micro Nutrient Powder (8-13 mg Fe, 200 µg Vit A, 20 mg Vit C, 20 µg folic acid, 5 mg Zn, 0.5 µg Vit B12 & 0.5 mg Vit B2) *Nutrition education*- Multi micronutrients	MNP and learning trial has a positive effect on the health and nutrition of the children.^20^
Children (5 to 15 years)	RCT	*Fortified food- *Fortified Salt (3000 IU of vit A, 10 mg Fe, 40 ppm I, 1 mcg of vit B12 & 100 mcg folic acid/10 g fortified salt) *Nutrition education-* Iron & vitamin A for 8 months	Improved the iron status and retinol status in the population.^28^

Abbreviations: RCT, randomized controlled trial; CRCT, cluster randomized controlled trial; RDBPCT, randomized, double-blind, placebo-controlled trial; DBCRCT, double blind cluster randomized control trial; DBRCT, double blind randomized controlled trial.

####  Food based approach -dietary food supplementation

 Food-based approach was aimed at correcting iron deficiency anaemia, through an randomized controlled tria focusing on increasing iron intake through dietary diversification among preschoolers for a period of 6 months.^21^ A short term intervention with garden cress seed enriched biscuits for 3 months revealed a favourable influence on the nutritional profile of malnourished and anaemic school children in Punjab when compared to the controls.^22^ The fortified salt with iron, iodine, vitamin B12, folic acid and zinc supplemented to women and children in Tamil Nadu caused a significant decline in the prevalence of anaemia and zinc deficiency with no change in iron deficiency anaemia compared to the controls.^23^

####  Food based approach -fortified food supplementation

 Biofortification of crops intends to improve the micronutrient status. In the double blind randomized controlled trial (6 months), biofortification of basic food crops with zinc was shown to be one of the cost-effective and sustainable strategy for combating zinc deficiency and preventing morbidity among Punjabi preschoolers.^24^ In Bangalore, children whose mothers received oral vitamin B12 (50g) supplementation had significantly higher expressive language scores than children whose mothers received placebo.^25^ Calcium supplementation was effective in improving the bone mineral density among premenarchal school girls based on the double-blind intervention (1 year) in Pune.^26^

####  Multiple intervention strategies- food-based approach & micronutrient supplementation

 The utilization of different techniques targeted and provided to various target populations characterizes multiple intervention programs. The combination of food-based and micronutrient supplements includes intervention studies utilizing local food sources and micronutrient supplements. Supplementation of Sorghum (*Sorghum bicolor*), a millet rich in micronutrients along with IFA supplements aimed at combating micronutrient deficiency among children (9-12 years). The above study was carried out in Andhra Pradesh showed significant results in improving blood haemoglobin, serum folic acid, albumin, retinol-binding protein, serum ferritin and serum calcium levels.^27^

####  Multiple intervention strategies- food-based approach & community-based approach

 In Tamil Nadu, one such integrated approach in combination with knowledge intervention and supplementation of fortified foods aimed at reducing micronutrient deficiencies. Nutrition education along with fortified salt with vitamin A, iron, iodine, vitamin B12 and folic acid proved to be effective in improving iron and vitamin A status among children (5-15 years). The health information was effective in improving their knowledge, diet choice skills and haemoglobin levels.^28^

###  Micronutrient interventions for adolescents

 Micronutrient deficiency has a worldwide health impact on adolescents since its manifestations are less evident and generally appear when the situation is severe and has already resulted in serious health issues. Nutrition education and counselling, micronutrient supplementation, food fortification and multiple micronutrient interventions are just a few of the nutrition-specific strategies that have been promoted to combat malnutrition. Micronutrient interventions that improve the micronutrient status of the target population are depicted in Table 4.

**Table 4:** Micronutrient interventions for adolescents

**Target population**	**Study design**	**Intervention**	**Findings**
**Food based approach -dietary food supplementation**			
Adolescents (10-14 years)	RCT	Millet-based mid-day meals (Iron: 40-50%, calcium: 60% and zinc: 40–50%)	Replacement of supplement rice with millets improved nutrient performance in school feeding programs.^29^
**Food based approach -fortified food supplementation**			
Adolescent boys & girls (12–16 years)	DBRCT	Biofortified pearl millet (21–52 ppm Fe) for 6 months	Improves iron status and several makers of cognitive performance.^30^
**Micronutrient supplementation**			
Adolescent girls (11-18 years)	RCT	100 mg Fe & 500 mcg folic acid	Significant reduction in the prevalence of anaemia.^31^
Young Adolescents	Placebo-RCT	2 μg/day vit B12 & 500 μg folic acid for 6 months	Increase the plasma vitamin B-12 concentrations & haemoglobin.^32^
**Community based approach**			
Adolescent girls	CS	*Micronutrient Supplements *-Iron & folic acid (100 mg Fe & 500 µg folic acid)	Knowledge centered approach was effective in scaling up of public health nutrition intervention in Anaemia Control Programme.^33^
Adolescents (12-15 years old)	RCT	*Dietary foods* -Fruits & vegetables for 3 months	The education awareness was effective in the consumption of fruits & vegetables.^34^
**Multiple intervention strategies- food based approach & micronutrient supplementation**			
Adolescent girls	RCT	*Dietary food-* Zinc enriched snacks *Supplements- *16.6 mg Zn for 10 weeks	Efficient to enhance the zinc, iron and vitamin A and C levels.^35^

Abbreviations: RCT, Randomized controlled trial; DBRCT, Double blind randomized controlled trial; CS, comprehensive survey.

####  Food based approach -dietary food supplementation & fortified food supplementation

 In Karnataka, the efficacy of adding millets to the mid-day meal programme had nutritional benefits on the health of the children compared to those who consumed fortified rice based mid-day meal.^29^ Another RCT study revealed that consuming iron-biofortified (21-52 ppm) pearl millet improves iron status and some measures of cognitive functions in adolescents of Maharashtra.^30^

####  Micronutrient supplementation 

 A few were studies concentrating on the adolescent population for decreasing the prevalence of anaemia. In Pune Rural Intervention in Young Adolescents (PRIYA) supplementation trial, the prevalence of anaemia knocks down to 59% with the administration of 2 µg/d vitamin B12.^31^ A community-based RCT trial in Delhi reported that the mean haemoglobin levels increased significantly after 6 months intervention with WIFS among adolescent girls.^32^

####  Community based approach 

 The Anaemia Control Programme (UNICEF) with a knowledge-centred approach scale up public health nutrition interventions and facilitate intersectoral convergence among different government departments and development partners to break the inter-generational cycle of undernutrition and deprivation.^33^ In a research study conducted in Tamil Nadu, parents were given awareness of the risks involved in frequent consumption of fast food, processed snacks, bakery products and carbonated beverages. Parents should also equally contribute on monitoring the diet of their children. Parent-teacher meetings highlighted the significance of the dietary and physical activity pattern along with their academic issues. This approach implemented was found to be effective in improving the dietary consumption of fruits and vegetables among early adolescents (12-15 years).^34^

####  Multiple intervention strategies- food-based approach & micronutrient supplementation 

 Zinc helps to release vitamin A from the liver, for the tissue metabolism and also zinc impairs iron absorption when given in a high dose. An RCT trial administered with a zinc-rich snack for adolescent girls in Pune along with zinc supplements (16.6 mg) impacted on improved zinc, iron, vitamin A and vitamin C parameters within a span of 10-week intervention period.^35^

 From the above literature, it was observed that the intervention through a combination of micronutrients showed significant results in the biochemical parameters rather than the single nutrient supplements. Nationalized intervention programs for micronutrient deficiencies such as WIFS forms the backbone of the majority of the research. Apart from the prenatal & postnatal interventions, the pre-adolescence and adolescent population show better improvement in the micronutrient status in terms of calcium and vitamin D.^25,36^

###  Micronutrient interventions for adults

 Anaemia is the major health issue among the adult population including both males and females. Intervention strategies have been adopted to combat micronutrient deficiencies to reduce the future metabolic issues in the below population. The strategies to alleviate micronutrient deficiencies is displayed in Table 5.

**Table 5:** Micronutrient interventions for adults

**Target population**	**Study design**	**Intervention**	**Findings**
**Food based approach -dietary food supplementation**			
Male & female adults	RCT	Dairy foods (0.99 µg Vit B12) for 14 days	Increased bioavailability of vitamin B-12 (dairy foods).^37^
Non-pregnant women	RCT	Green leafy vegetables (Iron) for 1 year	Improved the haemoglobin level.^38^
Preconception women	RCT	Green leafy vegetables-50.4%, fruits-15.5% & milk-50.4% (Iron, β-carotene, riboflavin, folate, vitamin B12 and calcium)	The intervention had no influence on the fetal size or growth ultrasonography measurements.^39^
**Food based approach- fortified food supplementation**			
Adult women (20-60 years)	RCT	Fortified soy biscuits (8.4 mg Fe & 120 µg folic acid)	Improved haemoglobin, serum iron and total white blood cell (WBC) count.^40^
Women	RCT	Fortified Salt (10 mg Fe, 400 µg I, 4 µg Vit B12, 100 µg folic acid & 10 mg Zn)	There was a significant decline in the prevalence of anaemia and zinc deficiency with no change in iron deficiency anaemia.^23^
**Micronutrient supplementation**			
Male & Female Adults	Prospective study- Iron	Ferric ammonium citrate (elemental iron 32.8 mg/tablet)/ferrous sulphate (elemental iron 46.8 mg/capsule/ferrous calcium citrate (elemental iron 25 mg/ tablet)/ferrous fumarate (elemental iron 50 mg/tablet) for 3 months	Four formulations improved mean hemoglobin and anemia indices without significant difference between groups.^41^

Abbreviation: RCT, randomized controlled trial.

####  Food-based approach -dietary food supplementation 

 A potential dietary strategy in Pune demonstrated that regular intake of milk containing 0.99 µg vitamin B12 for 14 days improved vitamin B-12 status among vitamin B-12 deficient vegetarians.^37^ Consumption of iron-rich foods such as green leafy vegetables increased from 44.7% to 60.6% after one year intervention among non-pregnant women in Pune which subsequently improved their haemoglobin level.^38^ In a food-based RCT executed among Indian women living in Mumbai slums, the daily micronutrient-rich snack consumption before conception and during pregnancy showed no influence on ultrasound measurements of fetal size or growth.^39^

####  Food based approach- fortified food supplementation

 An RCT experiment carried out in Tamil Nadu proved provision of fortified soy biscuits with iron and folic acid showed an appreciable change in hematological conditions of the cotton ginners who were exposed to cotton dust with a high prevalence of respiratory diseases, increased morbid pattern and anaemia.^40^ The fortified salt supplemented to women and children in Tamil Nadu for 8 months caused a significant decline in the prevalence of anaemia and zinc deficiency with no change in iron deficiency anaemia compared to the controls.^23^

####  Micronutrient supplementation

 In Kochi, adults with nutritional IDA showed improved mean haemoglobin and anaemia after 3-month supplementation with four different formulations of iron supplements (ferric ammonium citrate/ ferrous sulphate /ferrous calcium citrate /ferrous fumarate).^41^

###  Micronutrient interventions for pregnant women

 Poor nutrition during pregnancy has been related to adverse mother and child outcomes such as higher chances of infertility, abortion, foetal intrauterine growth restriction, and perinatal death. The nutritional deficiency in the pregnancy period give birth to underweight baby, stunted child, weak adolescent girl and again a malnourished mother in the intergeneration cycle. This section describes intervention trials that looked at the effects on improving the micronutrient status of pregnant women (Table 6).

**Table 6:** Micronutrient interventions for pregnant women

**Target population**	**Study design**	**Intervention**	**Findings**
**Food based approach -dietary food supplementation**			
Pregnant women	RCT	Vegetables, fruits, fish, cereals, nuts, seeds, spreads, cooking oils, sauces and snacks (15 mg/d Vit E)	The viability of food based intervention improved the consumption of dietary vitamin E.^42^
**Food based approach -fortified food supplementation**			
Pregnant Women (15-49 years)	Quasi-experimental design	Fortified wheat flour (3300 IU/kg Vit A, 1.5 mg/kg folic acid & 60 mg/kg Fe)	There was no impact on the haemoglobin level and anaemia reduction.^43^
Pregnant women (18–35 years)	CSS	Fortified salt (55·9 ppm & 18·9 ppm iodine) for 3 years	The iodized salt programme ensures that pregnant women get enough iodine and that their children have greater iodine consumption.^44^
**Micronutrient supplementation**			
Pregnant women	RCT	60 mg iron-folic acid for 6 months	Reduced mortality, preterm births & increased birth weight of infants.^45^
Pregnant women	Placebo RCT	50 μg Vitamin B12	Impact on infant cognitive outcomes.^46^
Pregnant women	CSS	5 mg/d folic acid & 15 mg/d Vit B12 for 2 years	Increase in ratio of folate to vitamin B12 concentration.^47^
Pregnant women (≥ 18 years)	RDBPCT	50 µg Vit B12	Vitamin B-12 status measurements in maternal plasma and breast milk have improved.^48^
**Community based approach**			
Pregnant women & their infants	CRCT	*Complementary foods*-Vitamin A, calcium, iron & zinc for 6 months	Complementary foods by ICDS programme improves dietary intake, length and mental development.^49^
Pregnant women	CSS	*Micronutrient supplements*- Iron	Anaemia was prominent in spite of the resources and awareness given.^50^
Pregnant women & their children	CRCT	*Micronutrient supplements*- Iron, folic acid, calcium & zinc * feeding practices, family planning, health services, hygiene, sanitation, girl’s education & women empowerment *for 2 years	Improves dietary diversity, hand washing, and infant survival, but does not improve micronutrient status in short term.^51^
Pregnant women, breastfeeding women & mothers with infants	Formative qualitative study	*Easy to use toolkit*	An easy-to-use toolkit as an appropriate intervention to enhance nutritional intakes of pregnant and lactating women, as well as children.^52^
Pregnant women and mothers with children (6–24 months)	KAP survey model	*Home gardens and backyard poultry *– Green leafy vegetables, Guava, Mango, Lime, beans, tomatoes, okra & eggs for 3 years	Home garden and back yard poultry helps in reduction in malnutrition in the children.^53^
**Multiple intervention strategies- food based approach & micronutrient supplementation**			
Pregnant women (17–40 years)	Cohort study	Dietary food -folate rich foods Supplements-1.2 mg/d Vit B12 & 5 mg/d folic acid	Vitamin B12-folate status improved the poor birth outcomes.^54^
**Multiple intervention strategies- community based approach & micronutrient supplementation**			
Pregnant women	Quasi-experimental design	*Supplements-* 150 mg Fe, 15 µg Vit B12 & 61.8 mg Zn *Nutrition education-* Multi-micronutrients for 3 months	The health awareness was effective in helping the pregnant women to improve their knowledge regarding anaemia in pregnancy.^55^
Pregnant Women (15-49 years	CSS	*Supplements-* 100 mg Fe & 500 µg folic acid *Nutrition education-* IFA supplements	Intervention was effective in improving better utilization of prenatal services.^56^

Abbreviations: RCT, randomized controlled trial; CRCT, cluster randomized controlled trial; RDBPCT, randomized, double-blind, placebo-controlled trial; CSS, cross-sectional study; KAP, knowledge attitude practices.

####  Food-based approach -dietary food supplementation & fortified food supplementation

 Clark et al^42^ observed that dietary intervention demonstrates as a feasible and acceptable form of food-exchange intervention to optimize dietary vitamin E intake during pregnancy (RCT).^42^ Introduction of fortified wheat flour (iron, vitamin A & folic acid) in Tamil Nadu and Punjab through the public distribution system resulted in an additional 8% decline in anaemia compared to the neighbouring states without wheat fortification programs serving as controls.^43^ A long term iodized salt programme for 3 years conveyed that adequate iodine intake was noticeable among pregnant women and their children in Karnataka.^44^Synergy of nutrients maximizes the efficacy of each individual nutrient in the combination to increase the bioavailability of other nutrients. Keeping micronutrients bioavailable as a key and implementing or developing good synergic foods helps to improve the overall micronutrient status of the population.^36^

####  Micronutrient supplementation

 The RCT trial on pregnant women showed that antenatal IFA supplements (60 mg iron-folate) improved female neonatal survival and improved birth outcomes for infants delivered to malnourished and anaemic mothers.^45^ Several studies suggested that iron, folate and vitamin B12 were the key micronutrients intervened among the pregnant population. Studies required a long intervention period from pregnancy to postpartum stage to examine the cognitive outcomes among children. Studies reveal that supplements (iron, vitamin B12 and folic acid) were initiated from the pregnancy period and the impact was assessed for the children aged 6 months. Based on the placebo RCT trial in Karnataka, there was a significant effect of vitamin B12 on the cognitive performances of the infants based on Bayley Scales of Infant Development-III.^46^ The CSS study carried out in Pune discussed the combined association of folate and vitamin B12 which increased plasma homocysteine, lowering of neonatal birth weight, birth length, head circumference and chest circumference among pregnant women.^47^ Oral supplementation of vitamin B12 (50 µg) to urban Indian women in Karnataka throughout pregnancy and early lactation significantly increases vitamin B-12 status of mothers and infants compared with placebo recipients.^48^

####  Community based approach

 Education-based materials used in the intervention over a period of 6 months helps to achieve behavioural change among pregnant women for improving dietary diversity and increased consumption of locally available foods particularly animal foods based on a randomized trial in Hyderabad. This cluster randomized controlled trial also checked how teaching on complementary feeding increases children’s dietary intake, growth and development.^49^ The CSS study conducted in Tamil Nadu showed that a preventable nutritional deficiency like anaemia still prevails if the knowledge intervention fails to address the myths and taboos during pregnancy.^50^ The CRCT study conducted in Jharkhand and Odisha for a period of 2 years (from third trimester of pregnancy to 18 months of childbirth) on the significance of micronutrient supplements, better feeding practices, family planning, health care services, hygiene and sanitation did not have an effect on maternal and child anthropometric outcomes whereas there was a significant improvement in dietary diversity, hand washing practices and child mortality status.^51^

 In Bihar, a novel feeding toolkit consisting of a marked bowl, slotted spoon and a matching visual counselling card is highly acceptable and can be used by families to enhance dietary practices of women during prenatal and the postpartum period as well as the amount and quality of feeding of small children.^52^An added interventional approach on educating pregnant mothers on better feeding practices showed a significant behavioural change in breastfeeding, introduction of pre-lacteal foods, myths and taboos during pregnancy, initiation and frequency of complementary feeding. The long-term strategy for 3 years in introducing homestead gardens and backyard poultry was effective in reducing malnutrition among children (6-24 months) in Telangana.^53^

####  Multiple intervention strategies- food-based approach & micronutrient supplementation 

 A cohort study in Bangalore supplemented Group I with vitamin B12 and folate-rich foods along with vitamin B12 and folate supplements (1.2 mg/d Vit B12 & 5 mg/d folic acids) and Group II with vitamin B12 and folate supplements only. This study showed that group I was effective in reducing adverse birth outcomes during pregnancy and a similar trend was observed in the analysis of blood micronutrient status.^54^

####  Multiple intervention strategies- community based approach & micronutrient supplementation

 The three-month quasi-experimental strategy used in Karnataka was helpful in assisting pregnant women in considerably improving their awareness of anaemia and capacity to select foods rich in iron, protein and vitamin C. Micronutrient supplements (iron, vitamin B12 & zinc) would further ensure to help in the increase of their haemoglobin level. In spite of micronutrient supplements, nutrition education paved way for understanding the significance of micronutrients with improved health-seeking behaviour.^55^ In all twenty-nine states and seven union territories of India, intake of IFA supplements and supplemental meals was predicted to improve with women’s education, household affluence, early and prenatal visits, according to the CSS design. Continuous monitoring and assessment of IFA supplements and supplemental meals supplied in targeted groups and communities is critical to increasing coverage and lowering the impact of undernutrition during pregnancy.^56^

 In this section, it was concluded that pregnant women and postnatal mothers could be addressed through micronutrient fortifications. Improved and innovative ways of providing micronutrients to target populations through suitable fortification techniques should be pursued. The problems in the implementation of food fortification programs involve many issues like safety, technological problems and cost estimations.^57^ There was no empirical evidence for the nutritional advantages of fortification of food with vitamin A, vitamin D and calcium for reproductive-age women, necessitating the need for future quality study on food fortification.^58^

###  Micronutrient interventions for lactating women

 Postpartum micronutrient deficit is connected with decreased quality of life, decreased cognitive capacities, emotional instability, and depression, forming a substantial health concern in postpartum women. Several strategies have been used to supply micronutrients to breastfeeding women. Education, dietary modification, supplementation, and fortification are examples of these tactics, which can be used alone or in combination is shown in Table 7.

**Table 7:** Micronutrient interventions for lactating women

**Target population**	**Study design**	**Intervention**	**Findings**
**Micronutrient supplementation**			
Lactating mothers	RCD	20 mg Fe & 100 mcg folic acid	Helps in preventing paediatric anaemia.^59^
Lactating mothers	DBRCT	10 mg/d Zn	Cobalamin concentration enhances zinc intake in children.^60^
Lactating mothers	RDBPCT	1.8 μg Vit B12 & 150 μg folic acid	Improves the neurodevelopment & cognitive function.^61^
Lactating mothers	Placebo-RCT	50 µg Vit B12 for 6 months	Positive association on cognitive outcomes in children.^62^
**Community based approach**			
Mothers with children under age five	Quantitative approach and descriptive survey design	*Micronutrient supplements*-Vitamin A, vitamin D and iron	There is a significant association between knowledge and the source of information gained through IEC materials.^63^
**Multiple intervention strategies- food based approach & micronutrient supplementation**			
Lactating mothers & their infants	RCT	*Dietary food -*Choco Energy Bites, Wheat flour-cumin sweet *( Panjeeri )*, Cumin *(Jeera) *crackers & Nut mixture *Supplements-*Multivitamins, (iron, zinc, iodine, selenium and copper)	The micronutrient snacks and supplements improved the maternal nutritional status.^64^

Abbreviations: RCT, randomized controlled trial; RDBPCT, randomized, double-blind, placebo-controlled trial; DBRCT, double blind randomized controlled trial; RCD, randomized crossover design; CSS, cross-sectional study.

####  Micronutrient supplementation 

 The randomized crossover design study showed that both iron products (MNP and IFA supplements) supplemented to the mothers was an acceptable policy option for preventing paediatric anaemia among children 6–23 months in Bihar.^59^ Multiple micronutrients play a significant role in micronutrient supplementation. In Haryana, the double-blind trial among mothers of 6-30 months old children with low cobalamin status showed significant benefit in their children when taken vitamin B12 along with zinc supplementation (10 mg/d).^60,61^ The RDBPCT trial of oral vitamin B12 (1.8 μg vitamin B12 & 150 μg folic acid) supplementation among mothers resulted in improved neurodevelopmental outcomes and growth in their children.^62^

####  Community-based approach

 Through a quantitative approach and descriptive study design the knowledge and awareness on vitamin A, D and iron were imparted for mothers of children below 5 years in Tamil Nadu. A significant association was shown between knowledge and the source of information gained through IEC materials.^63^

####  Multiple intervention strategies- food-based approach & micronutrient supplementation

 Postnatal mothers supplemented with micronutrient-rich snacks (Choco Energy Bites, Wheat flour-cumin sweet *( Panjeeri )*, Cumin *(Jeera) *crackers & Nut mixture) along with multi vitamin supplements during their first 6 months improved maternal body mass index (BMI), mid-upper arm circumference (MUAC), haemoglobin concentrations in both mothers and infants. Awareness and counselling on better feeding practices and infant care resulted in improved exclusive breastfeeding practice among mothers.^64^

## Discussion

 Recorded infant interventions have been designed to improve the behavioral, cognitive and physical development of the child. Fortified complimentary micronutrient powder supplementation and awareness among mothers of the children help to increase the micronutrient status of the infants. Selected intervention studies in preschoolers confirm the importance of nutrition requirements for their rapid growing phase. School-based nutrition interventions with inclusion of antioxidant rich fruits were designed to improve dietary intake among the children. Effective results were reported by multiple interventions encompassing nutrition education, behavioural interventions and other complementary strategies including dietary supplementation, fortification and micronutrient supplementation.

 A focus on adolescent intervention is important not only to improve the health status of women but also to ensure optimal foetal growth and development to prevent the vicious cycle of intergenerational transmission of undernutrition. Recent studies evidently proved that approaches with dietary supplementation and cost-effective fortified foods targeted on adolescent age group improved their nutritional status. The period of pregnancy is considered to be the most critical biological period in women’s lifespan considering the birth outcomes and women’s health. Intervention studies revealed that fortified foods and the use of recommended dietary supplements remains the preferred means for meeting dietary requirements for micronutrients during pregnancy and lactation.

## Conclusion

 Governmental and Non-Governmental Organisations including research institutions, have implemented various strategic intervention plans to address micronutrient deficiencies in Indian states. Food-based intervention can be enhanced using modern technologies to develop cost-effective, enriched and fortified food utilizing locally available food resources. The majority of the micronutrient supplementation research interventions were focusing to cover pregnant women and children through various nationalized programs on iron and folic acid to address IDA. Knowledge interventions were proved to be more effective when combined with other intervention methodologies than stand-alone. Multiple interventions are effective that could lead the road to innovations in approaches with diverse dietary intake, developing multiple micronutrient supplements, fortifying foods and nutrition interventions to address calcium, zinc, iodine, vitamin D and vitamin A deficiencies among the vulnerable population.

 Future research could plan and strengthen the community-based knowledge intervention via participatory learning methods at the doorsteps with available social health activists, health care workers and volunteering research groups to make it more effective. Further additional strategies must achieve behavioural change for improving dietary diversity and increased quantities of locally available foods. Evidence-based multiple intervention studies covering a large population, on a long-term cross-sectional, vulnerable population is the need for the hour to design policies and programs for improving the micronutrient status of the community.

## Acknowledgements

 The author expresses heartful gratitude to Tribal Center of Excellence at Amrita Vishwa Vidyapeetham.

## Authors’ contributions

 Concept: JR. Study design: SH and TA. Systematic search: SH. Critical reviews: SH, JR and TN. Writing: SH. All authors had primary responsibility for the final content of the manuscript and read and approved the final manuscript.

## Funding

 No funding was received to assist with the preparation of this manuscript.

## Ethical approval

 As a narrative review, additional approval from ethical committee was not applicable.

## Competing interests

 The authors declare no conflict of interest.
